# Adapting the Germ Defence Web-Based Intervention to Improve Infection Prevention and Control in Care Homes: Interview Study Among Care Home Staff

**DOI:** 10.2196/66706

**Published:** 2025-02-19

**Authors:** Alex Hall, Johanna Aguilera-Muñoz, Lisa McGarrigle, Charlotte Eost-Telling, James Denison-Day, Christie Cabral, Merlin Willcox, Chris Todd

**Affiliations:** 1 School of Health Sciences University of Manchester Manchester United Kingdom; 2 Manchester Academic Health Science Centre Manchester United Kingdom; 3 National Institute for Health and Care Research Applied Research Collaboration Greater Manchester Manchester United Kingdom; 4 Centre for Clinical and Community Applications of Health Psychology University of Southampton Southampton United Kingdom; 5 Centre for Academic Primary Care University of Bristol Bristol United Kingdom; 6 Department of Primary Care and Population Sciences University of Southampton Southampton United Kingdom

**Keywords:** care homes, long-term care, nursing homes, infection prevention and control, behavioral intervention development, person-based approach, qualitative

## Abstract

**Background:**

Infection prevention and control (IPC) is vital in care homes as it can reduce morbidity and mortality by 30%. Ensuring good IPC practice is a perennial challenge in the varied and complex context of care homes. Behavior change interventions delivered via digital technology may be effective in improving IPC among care home staff.

**Objective:**

This study aimed to evaluate how an evidence-based, digital behavior change intervention called Germ Defence can be rapidly adapted to meet the needs of care homes.

**Methods:**

This study applied the person-based approach, which emphasizes iterative approaches to optimizing interventions via individual user feedback. Phase 1 involved initial edits to the website by the research team to create Germ Defence for Care Homes (GDCH) version 1. Phase 2 consisted of stakeholder consultation on GDCH version 1 followed by edits to create GDCH version 2. The formal research (phases 3 and 4) involved individual think-aloud interviews with 21 staff members from management, care, and ancillary positions in 4 care homes providing real-time feedback as they worked through GDCH. Edits were made to create GDCH version 3 between phases 3 and 4. During the development of GDCH versions 2 and 3, it became clear that the intervention would need more fundamental changes beyond the pragmatic, incremental changes that would be possible within the scope of this study. Analysis was completed via a rapid, qualitative descriptive approach to develop a high-level summary of key findings from the interview data.

**Results:**

There were mixed results about the attractiveness of GDCH and its suitability to the care home context. Participants felt that the images needed to be aligned much more closely with the meaning of adjacent text. Many participants felt that they would not have time to read a text-based website, and some suggested that more engaging content, including audio and video, may be preferable. Most participants felt that the overall concept of Germ Defence was clearly relevant to their context. Some felt that it might be a useful introduction for new staff members or a refresher for current staff, but others felt that it did not add anything to their existing IPC training. There were mixed opinions about the level of detail provided in the information offered by the site. While the goal-setting behavior change mechanism may have potential, the findings suggested that it may be unsuitable for care homes and more work is needed to refine it.

**Conclusions:**

Much more work needs to be done to make Germ Defence more engaging, accessible, and relevant to the care home workforce. Our study highlights the challenges of rapidly adapting an existing intervention to a new context. Future research in this area will require a pragmatic methodological approach with a focus on implementation.

## Introduction

### Background

Infection prevention and control (IPC) is vital in long-term care facilities as it can reduce morbidity and mortality by 30% and may reduce unplanned hospital admissions and overuse of antibiotics [1]. In England (the setting of this study), IPC is a central focus of the Care Quality Commission, the independent health and care regulator that inspects long-term care facilities [2]. International terminology on long-term care facilities varies; in this paper, we use the term *care home* to refer to a long-term care facility providing 24-hour care, either with nursing (ie, in which residents with complex needs receive care provided by registered nurses on-site) or without nursing (ie, in which residents with less complex needs receive support with personal care from nonregistered staff, with nursing and health care input provided as required by community practitioners). In England and Wales, there are approximately 278,000 care home residents aged ≥65 years (approximately three-quarters of whom are aged ≥80 years), approximately half of whom live in homes with nursing and half of whom live in homes without nursing and over two-thirds of whom experience substantial limitations in their ability to carry out day-to-day activities [3].

The COVID-19 pandemic of 2020 to 2022 brought both the importance of and challenges in ensuring good IPC to the forefront of public consciousness [4-6]. It also raised the profile of care homes, which are often marginalized and forgotten by the public and policy makers and which were hit particularly hard [7]. Many countries struggled with ensuring good IPC in care homes during the pandemic, but although international comparisons are difficult [8], the United Kingdom is perceived to have fared particularly badly. In England, the first 5 months of the pandemic saw approximately 20,000 excess deaths among care home residents that were directly attributable to COVID-19 [9]. Before the pandemic, care home residents in England aged ≥65 years had a 10-fold higher mortality than older people in private homes, but in the first wave of the pandemic, this rose to an 18-fold difference [10]. At least for the first months of the pandemic, care homes experienced substantial, widely reported challenges in accessing COVID-19 testing and in isolating residents and a lack of availability of personal protective equipment (PPE; eg, gloves, face masks, and aprons) [11,12]. Other systemic problems linked to a higher risk of COVID-19 outbreaks included a lack of statutory sick pay for staff (thereby discouraging staff to take time off work) and reliance on agency workers (who work across multiple sites) to address workforce shortages [13]. Care homes were locked down with only essential staff access, and government guidance on visiting care homes was not issued until July 2020, which was 4 months into the pandemic, and, thereafter, proceeded to change frequently. Care home managers struggled with late notice about changes (often being informed after national televised briefings), gaps regarding risks and harms to residents, a lack of clarity on how to interpret guidance coupled with uncoordinated support from local regulators, and lack of acknowledgment of implementation challenges [14]. Restrictive IPC measures in care homes have been the subject of intense debates regarding their psychosocial impacts on residents and potential breaches of mental capacity and human rights law [15,16].

COVID-19 stands as the contemporary paradigmatic case study of (failures in) IPC in care homes, but many of the challenges it highlighted long predate the pandemic. Other common viral infections in care homes include seasonal influenza (another respiratory virus) and norovirus (the main cause of gastroenteritis, which is spread through physical contact); bacterial infections such as *Salmonella* sp and group A *Streptococcus* sp; and infections caused by nonviral and nonbacterial agents, including scabies [17-19]. Care homes are particularly vulnerable to all these pathogens because of the physical proximity between people and the fact that most residents are of advanced age and living with chronic health conditions and require hands-on work with physical contact. In England, all care homes are required to have IPC plans, and all staff are required to complete IPC training [2]. A Cochrane rapid review of qualitative evidence (published between 2005 and 2020) of barriers to and facilitators of health care workers’ adherence to IPC guidelines showed numerous enduring challenges that resounded during the COVID-19 pandemic, including (but not limited to) lengthy and changing guidance, increased workload in following guidance, lack of easy access to handwashing facilities, lack of training about infections and PPE use, lack of good-quality PPE, and difficulties in using PPE if patients felt frightened or stigmatized by it [20]. Some of these challenges are particularly pertinent to care homes as they function as both people’s homes and places of care and are staffed by a workforce that may lack training and skills compared to those in other health care sites, including key ancillary staff (eg, cooks and cleaners) who are crucial to IPC [21]. Hand hygiene among care home staff may have been poor for many years [22], and a systematic review of evidence published before the pandemic found that poor hand hygiene was the most common cause of transmission of infections in care homes [23]. The COVID-19 pandemic reinforced the importance of PPE and behavioral measures such as handwashing to reduce transmission of infection [24] but also strikingly highlighted unintended consequences where use of PPE such as gloves may actually reduce compliance with handwashing [25]. A recent meta-ethnography of qualitative studies of IPC in care homes found that IPC can be considered by staff to be “outside” the control of the care home both in terms of the source of infection and in actions to control it—the tension of implementing IPC in what is meant to be a homely environment appears to contribute to a sense that outbreaks are inevitable, with care home staff ambivalent about the benefits of applying IPC and perceiving a lack of ownership over IPC practice [26]. Ensuring good IPC practice is a perennial challenge in the varied and complex context of care homes. It remains of primary importance to consider how to improve IPC interventions and their implementation to help protect care home residents and staff from outbreaks of infectious diseases.

A recent systematic review of randomized controlled trials of interventions to prevent transmission of infections in care homes found only 4 trials of interventions to improve hand hygiene and IPC, with variable results [27]. Only 1 study showed a reduction in pneumonia incidence, 1 showed a reduction in influenza outbreaks, and 1 showed a reduction in incidence of influenzalike illness. Achieving high levels of adherence to infection control is challenging, and the interventions were all different; 3 contained in-person presentations, and only 1 contained an e-learning component. The review recommended developing more impactful behavior change interventions for improving IPC in care homes using behavioral science and the person-based approach.

### Germ Defence: A Web-Based Behavior Change IPC Intervention

The World Health Organization outlines 8 core components of IPC programs in health care, including emphasis on multimodal strategies to change individual behavior [28]. A systematic review of IPC interventions in care homes found that those incorporating behavior change strategies via education, monitoring, and feedback were the most successful [29]. There is increasing recognition of the potential role of digital health technologies in IPC [30-33], which may be associated with their increased use to facilitate behavior change across a range of health domains, particularly via goal setting and self-management [34]. The intervention in this study is Germ Defence, a mobile-friendly website providing targeted, tailored advice on how and why people should adopt IPC behaviors, supplementing public health guidance with evidence-based and theory-based behavior change techniques [35]. It consists of introductory content about the importance of IPC followed by a series of pages arranged in parallel pathways where users can select from 2 components of interest (handwashing and reducing illness) and follow sequential pages of content streams that result in tailored advice and goal setting to improve or increase IPC behaviors.

Germ Defence was originally developed in response to the swine influenza (H1N1) and H5N1 influenza outbreaks of 2009 to 2010 in the form of an intervention then called PRIMIT (Primary Care Trial of a Website-Based Infection Control Intervention to Modify Influenza-like Illness and Respiratory Infection Transmission) to encourage handwashing delivered in 4 weekly sessions [36,37]. A subsequent large randomized controlled trial of >20,000 people living in shared households conducted across 3 winters (2011-2013) showed that the PRIMIT intervention increased handwashing and reduced respiratory tract infections [38]. The intervention was developed using the person-based approach, which involved conducting in-depth qualitative research to develop detailed understandings of how to overcome the behavioral barriers that users might encounter to engaging with the target behaviors [39]. It also used behavior change approaches to incorporate education, personalized goal setting, environmental prompting, and emotional motivation [40]. A process evaluation showed that the intervention was effective for both men and women, older and younger people, and people with both lower and higher levels of education [40]. Importantly, the biggest change in handwashing behavior occurred after the first of the 4 weekly sessions, so the intervention structure was subsequently adapted for wider dissemination to comprise 1 stand-alone session, with further optional content available immediately afterward [40]. The adapted intervention—now known as Germ Defence—was disseminated “in the wild” to help reduce seasonal colds and influenza.

In 2020, Germ Defence was rapidly adapted for the COVID-19 pandemic by a team of medical, public health and behavior change experts, and public contributors [35], with cross-sectional work highlighting its potential to improve IPC behavior [41]. It was endorsed by the chief medical officer for England as a priority via a large cluster-randomized controlled trial in primary care rolled out across all general practices in England (N=6579) [42]. At the same time, work with schoolchildren, parents, and school staff to use Germ Defence to mitigate the spread of COVID-19 in schools suggested that it is possible to adapt the intervention for use in this institutional context [43,44]. The sense of collective responsibility promoted by the intervention was seen to be persuasive in adopting the suggested behaviors, although lack of physical space and resources within schools may be barriers to adoption [43].

### Study Aim

IPC is a perennial challenge in care homes. Behavior change interventions delivered via digital technology may be effective in improving IPC. The existing evidence base for Germ Defence, its positioning as a national priority response to COVID-19, and its potential for adaptation to institutional contexts highlighted that consideration of adapting it to suit care homes was merited. The aim of this study was to evaluate whether and how Germ Defence might be adapted to meet the needs of care home staff.

## Methods

### Design

**Figure 1:**
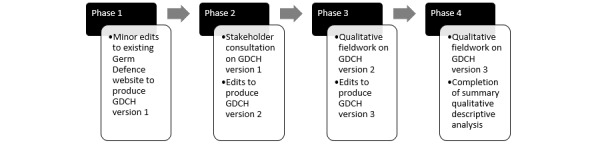
Four phases of the study.

This study followed Medical Research Council guidance on intervention development by drawing on the intervention development aspects of the person-based approach, which emphasizes iterative approaches to optimizing interventions [39,45,46]. It involved modification of the existing Germ Defence for Schools website [44] across 4 phases (Figure 1). We chose the school version as our starting point because of its potential greater initial applicability to an institutional context than the original version intended for use only in private residences. After its initial general introduction pages, the school version contains 2 pathways of advice that users can choose to work through, one focusing on how to protect themselves in their own homes and one focusing on how to protect themselves in school. We intended to mimic this structure and change the organizational pathway from schools to care homes. Given the limited scope of our project, we focused our attention on the general introduction pages and the organizational pathway only.

Phase 1 involved preliminary research team edits to the website to create Germ Defence for Care Homes (GDCH) version 1. Phase 2 consisted of stakeholder consultation followed by further edits to create GDCH version 2. Phase 3 was the first half of the formal research, involving an initial round of qualitative fieldwork with care home staff followed by edits to create GDCH version 3. Finally, phase 4 was a second round of qualitative fieldwork exploring care home staff perceptions on GDCH version 3.

### Preliminary Work: Phases 1 and 2

In phase 1, we made some initial small changes to the existing website to ensure that it referred to care homes rather than schools and to viruses more generally rather than to SARS-CoV-2 specifically. This became GDCH version 1. In phase 2, we held brief individual consultations with 8 staff members from 3 care homes (n=3, 38% managers; n=3, 38% carers; and n=2, 25% nurses) to gauge their initial responses to GDCH version 1. The changes they suggested were incorporated into GDCH version 2, which was then used in the qualitative fieldwork. Details of key changes are presented in the Results section.

### Formal Research: Phases 3 and 4

#### Recruitment

The formal research involved 2 phases of qualitative fieldwork with iterative intervention development. We conducted fieldwork with care home staff in Greater Manchester. We aimed to purposively sample between 4 and 6 care homes representing a range of characteristics known to be related to infection rates (large and small size, modern purpose-built and retrofitted houses, with and without nursing care, not for profit and for profit, and inspection quality rating) [9]. Within each care home, we aimed to recruit 5 to 6 staff members purposively sampled to represent a wide range of roles, including managers, carers, and ancillary staff. Therefore, the total estimated sample was between 20 and 36 staff members, which aligns with previous work on qualitative sampling [47]. Care homes were recruited via a range of existing networks and contacts in the region, including the National Institute for Health and Care Research Applied Research Collaboration Greater Manchester Care Home Research Collaboration and the Enabling Research in Care Homes network. Within each care home, we provided promotional materials about the study for display and sought guidance from managers as to suitable staff members to invite.

#### Data Collection

The person-based approach to intervention development involves using “think-aloud” interviews to understand users’ contexts and views on every aspect of the intervention and modifying the intervention accordingly [46]. Think-aloud interviews have roots in cognitive psychology and are commonly used to collect real-time feedback and information about people’s thought processes as they engage with a particular phenomenon of interest [48]. All staff members in phases 3 and 4 participated in individual think-aloud interviews. Participants were shown the contemporaneous version of the GDCH website and were asked to work through the intervention in real time. The think-aloud element drew on an interview guide that included neutral prompts and follow-up probes to encourage participants to elaborate on their actions and verbalizations in the moment (eg, “Can you tell me a bit more about that?”). This approach offered participants the opportunity to express immediate reactions to all aspects of intervention content and functionality as they used them, page by page. Details of key changes after each phase are presented in the Results section.

All interviews were conducted individually face-to-face on care home premises, lasting 28 to 54 minutes. They were audio recorded and transcribed verbatim. We also collected demographic data on participants’ age, ethnicity, role, and length of time in the post. Data collection was undertaken by one researcher (JA-M) in May 2023 to June 2023. This researcher had a background in qualitative research within organizational psychology and, before data collection, attended training on the person-based approach and think-aloud interviews.

#### Data Analysis

Our initial approach to data analysis was to adopt the “table of changes” analysis commonly used with the person-based approach [39,45,46]. This analysis offers a rapid but rigorous method for extracting quotes and feedback about each component of the intervention from the think-aloud interviews and considering the relative importance of possible changes to the intervention. One researcher (JA-M) read the interview transcripts and extracted quotes from them into a Microsoft Excel (Microsoft Corp) document detailing positive and negative comments by website page or section. This document was shared with the wider research team, and we discussed possible changes and their potential importance. Following phase 3, we made changes to GDCH version 2 that we considered important yet practicable within the resources of the study. The revised GDCH version 3 was then used in phase 4 with the same intention. However, during the development of GDCH versions 2 and 3, it became apparent that the website would need more fundamental changes than any of the pragmatic, incremental changes identified via the table of changes approach, which would be beyond the limited scope of this study. Therefore, we conducted a rapid qualitative descriptive analysis [49] to present a high-level summary of the key findings from the interview data. This involved taking the data that we had arranged in our table-of-changes Microsoft Excel documents, coding them, and grouping these codes into topic summaries. One researcher (AH) developed a set of codes that were discussed and agreed upon with the team. These codes (n=28) were applied to the remaining data by another researcher (JA-M). The team agreed on the grouping of these codes into 3 higher-level topics.

### Ethical Considerations

Research participants in phases 3 and 4 provided written informed consent. Ethics approval was granted by the University of Manchester Proportionate University Research Ethics Committee (November 8, 2022; reference 2022-15380-25722). Participants were informed about the study’s purpose and procedures and their right to withdraw at any time without detriment. To protect participants’ privacy, all data were fully anonymized. Research team members (JA-M and AH) reviewed the transcripts and removed any identifying information. Care homes were offered reimbursement of up to £25 (US $24.76) per staff member released to participate for up to an hour [50]. Payments were offered to the organizations rather than to individual staff members to contribute to backfilling of time if necessary and to avoid any perception of unfairness among nonparticipating staff.

## Results

### Care Homes and Participants

In total, we recruited 4 care homes. All homes were for-profit organizations, with variation in type of home, building design, size, and quality rating (Table 1).

Overall, we recruited 21 members of staff (Table 2). Participants occupied all major positions commonly found within care homes, including caring, senior caring, nursing, leadership and management, directorship, cleaning, and kitchen roles.

**Table 1:** Care homes in the study.

Care home number	Care home type	Building type	Size	Ownership	Inspection rating^a^
1	With nursing	Purpose built	26 beds	For profit	Good
2	Without nursing	Purpose built	40 beds	For profit	Outstanding
3	With nursing	Purpose built	31 beds	For profit	Requires improvement
4	Without nursing	Retrofitted house	17 beds	For profit	Inadequate (under the previous provider; was being reinspected at the time of this study)

^a^Care homes in England are subject to regular, usually unannounced inspection by the Care Quality Commission, the sector regulator. The inspection focuses on 5 domains assessing the extent to which a home is safe, effective, caring, responsive, and well led. The inspection results in 1 of 4 overall ratings: “outstanding,” “good,” “requires improvement,” or “inadequate.” Homes with the 2 lowest ratings are likely to be reinspected sooner than homes with the 2 highest ratings.

**Table 2:** Participant details^a^.

ID	Care home	Role	Study phase
P1	Home 1	Housekeeper	3
P2	Home 1	Senior carer	3
P3	Home 2	Health care assistant (enhanced)	3
P4	Home 2	Registered manager and director	3
P5	Home 2	Care assistant (plus domestic team lead)	3
P6	Home 2	Health and development lead	3
P7	Home 1	Senior carer	3
P8	Home 1	Deputy manager	3
P9	Home 2	Quality and compliance lead	3
P10	Home 2	Care team lead (plus acting care manager)	3
P11	Home 3	Carer and health care assistant	4
P12	Home 3	Carer and health care assistant	4
P13	Home 3	Domestic cleaner	4
P14	Home 3	Head chef	4
P15	Home 3	Team leader	4
P16	Home 3	Staff nurse (mental health)	4
P17	Home 4	Domestic assistant	4
P18	Home 4	Chef	4
P19	Home 4	Senior care assistant	4
P20	Home 4	Care assistant	4
P21	Home 4	Registered manager	4

^a^Time in the post ranged from 4 months to 21 years. The mean age was 43.38 (SD 13.27) years. In total, 90% (19/21) were female, and 67% (14/21) were of White British ethnicity.

### Phases 1 and 2: Minor Edits

**Figure 2:**
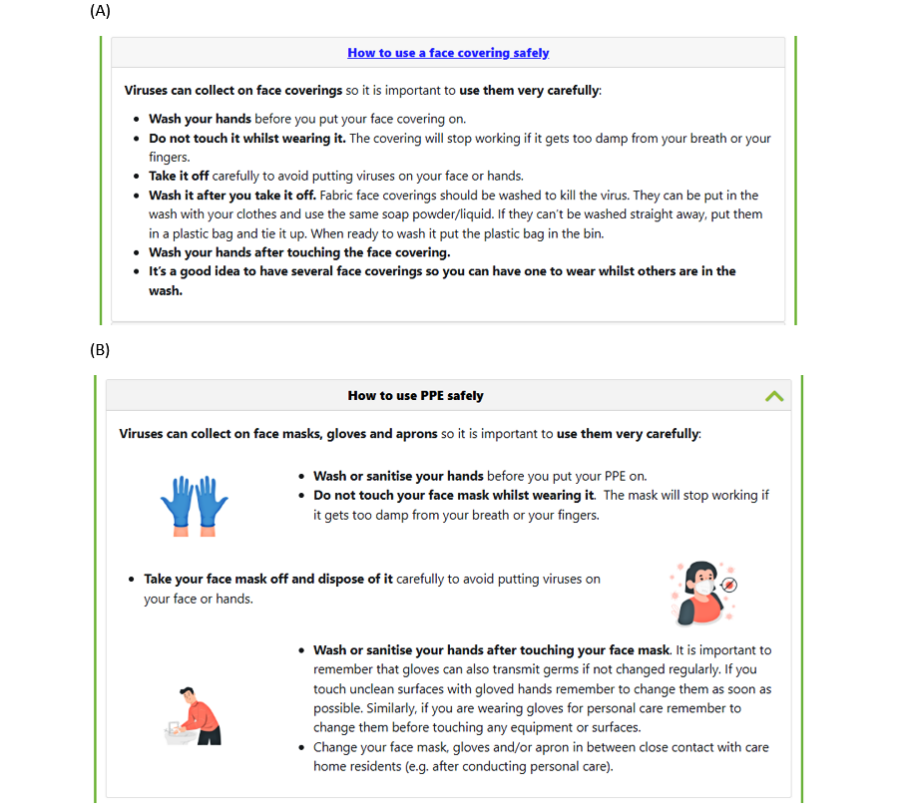
(A) Germ Defence for Care Homes version 1 face covering and (B) Germ Defence for Care Homes version 2 personal protective equipment (PPE) sections following stakeholder feedback in phase 2.

In phase 1, we made 2 types of minor changes to some of the wording of the website to create GDCH version 1. The first was to change references from “school” to “care home.” The second was to broaden the focus from COVID-19 to talk about infectious diseases more generally, including changing references to “COVID-19” to “respiratory viruses.” A major comment by the stakeholders consulted in phase 2 highlighted that a lot of the content in the section about mask wearing was very much dated to the COVID-19 pandemic, which may be unsuitable because permanent mask wearing was now not part of mandatory practice in care homes. When developing GDCH version 2, we amended this section to refer to using PPE more generally, which included some mention of masks but also other PPE commonly used in care homes, such as gloves and aprons, and delivering personal care (Figure 2).

In phase 1, GDCH version 1 retained the original Germ Defence behavior change component that asked participants to indicate how often they had performed certain IPC actions (eg, handwashing) per day over the previous week on an ordinal scale from 1 to 10 and then to make a plan for how frequently they intended to perform this behavior over the following week. In phase 2, some stakeholders felt that the scale from 1 to 10 would be unsuitable in the care home context as care home staff would wash their hands much more frequently. Therefore, in GDCH version 2, we removed this scale altogether. GDCH version 1 also contained a component that invited staff to think about points at which they had performed certain IPC actions in a typical working day on a 5-point scale (“almost never,” “sometimes,” “quite often,” “very often,” and “always”). We revised it to include more specific care home scenarios (eg, before coming into close contact with a resident and when donning PPE). We retained a sixth option of “does not apply to me” on certain questions (eg, those about PPE) because we were aware that some staff members, such as kitchen staff, would not perform certain activities, such as administering personal care.

### Phases 3 and 4: Key Findings and Further Intervention Modifications

#### Overview

Findings from phases 3 and 4 were grouped into 3 broad topic areas presented in the following sections along with key iterative changes made during the different project phases. *Attractiveness and accessibility of Germ Defence* reflects the overall appeal of the website and how accessible and engaging it was perceived to be. *Usefulness of Germ Defence* reflects the extent to which participants felt that Germ Defence might be useful in the care home context. *Individual behavior change in a complex environment* reflects findings relating to the goal-setting behavior change mechanism underpinning the intervention.

#### Attractiveness, Accessibility, and Usability of the Germ Defence Website

**Figure 3:**
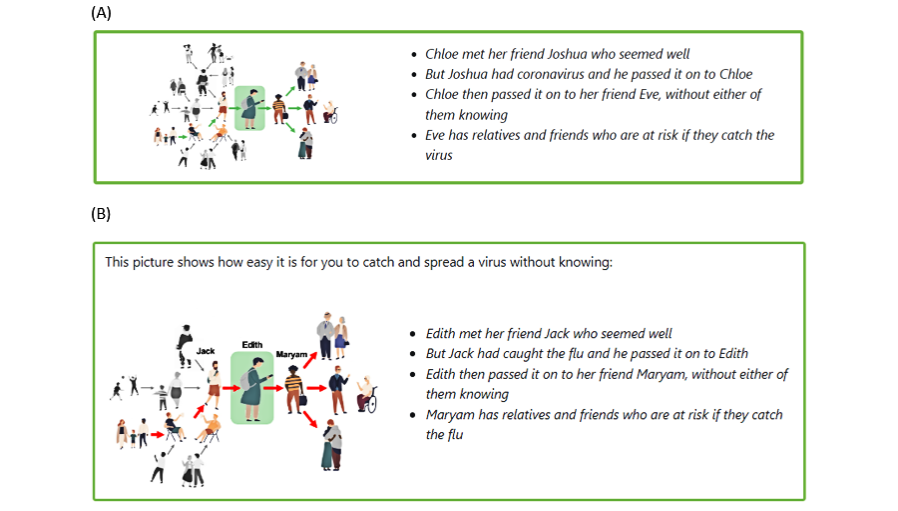
Cartoon showing how germs may spread between people (A) in Germ Defence for Care Homes (GDCH) version 2, used in phase 3, and (B) in GDCH version 3, used in phase 4, with character names changed.

Participants in phases 3 and 4 made a wealth of suggestions for revising the images and the text of the website. One example of this was in relation to a cartoon showing how germs may spread between people (Figure 3A). In phase 3, some participants found this cartoon confusing:

You’re looking at that thinking, I don’t know what’s going on. You’re reading that and then you’ve got to work out who’s who and which way it’s all going.

P6; health and development lead; phase 3

However, others were positive about the cartoon and its accompanying text:

...it just shows how easily it’s passed on to multiple other people. So, I think the text is enough and the picture is enough and I think they work quite well together.

P4; registered director and manager; phase 3

I think that tells the story quite well. Obviously, if it was in a care home setting...if you change the name Chloe to, like...Maureen or Beryl or Joyce, these are, sort of, the names that we have that are, sort of, older names for people...But, yeah, I mean, in terms of the image, I think it works.

P9; quality and compliance lead; phase 3

As an initial change following phase 3, we edited the names of some of the characters to be more reflective of care home resident age profiles (Figure 3B). Feedback in phase 4 suggested that the accompanying text was still necessary as the image alone did not clearly convey the message:

Once you read all this, you know what it is. But without the reading and without seeing anything, I can’t tell with this picture what it is.

P15; team leader; phase 4

Some participants suggested incorporating an image of a germ within the cartoon showing how it might move between people. Others suggested using color coding, such as red hands on one person and red shoulders on someone else they had touched. Another suggested adding Venn diagram–like circles around the images to show overlapping relationships. These suggestions highlighted that even revising this one cartoon may require substantial work, likely conducted over multiple phases of iterative development.

In both phase 3 and phase 4, some participants felt that they would not have time to sit and read through a text-based website. Some suggested that more engaging content, including via audio and video delivery, would be preferable (and identified some existing resources) and might address concerns surrounding English-language reading ability:

It’s a good way of explaining it, but with it being a website...There’s a video that we watched in training...I think it’s a hospital setting and he’s walking through, and it just shows how easy it is once you’re touching it and then that’s infected. I feel like that, from my experience, seeing that explains it a lot better than the picture there.

P5; care assistant and domestic team lead; phase 3

Or one thing you could add, I don’t know, is you could add an audio option where people could just click the audio and listen instead. You know, some people can’t understand English or they don’t...they can’t read so that’s another thing you can add to it.

P11; carer and health care assistant; phase 4

Regarding engagement with the website, some participants in phases 3 and 4 expressed a preference for practical training over reading material:

So, I think having that training face-to-face really enables people to see the risks that they’re putting themselves at if they’re not following the correct procedures and risks to our residents.

P4; registered manager and director; phase 3

I think practical’s better for me, personally, because academically, I don’t enjoy that side of it, I’m more practical, ’cause I’m a chef, I’m creative and that side.

P18; chef; phase 4

These findings highlighted that any adaptation of Germ Defence may need to include a practical element of applied action.

In phase 4, some participants suggested that, overall, the images on the web pages needed to stand out from the text more and cautioned that they needed to convey the message without depending on accompanying text as the level of English-language ability among the care home workforce is highly variable:

...a lot of the girls, we do it on iPads, we do it on phones, things like that. So that picture’s going to be minute...So I think you’re best putting that picture on a page on its own, if you want it to stand out.

P18; chef; phase 4

...we have people that might not be able to understand English as well as other people but they can understand what images are. So, that’s it, so this picture is equally as important as the contents within the text, yes.

[P11; carer and health care assistant; phase 4]

In summary, participants made a wealth of suggestions for changes that were not always mutually compatible. Incorporating these suggestions would require many phases of iterative development that were beyond the scope of our study.

#### Usefulness of Germ Defence Within the Care Home Context

The revised content about PPE (Figure 2) was received positively by some participants in phases 3 and 4. Some participants suggested that the content could better reflect requirements in the care home context, including how to properly dispose of PPE rather than simply saying, “dispose of it carefully.” Some acknowledged that mask wearing was not mandatory practice and suggested that masks were not used at all in their care homes:

I think it’s really good...I suppose, so mask wearing is becoming less prevalent in homes, so we wouldn’t...it wouldn’t be the sort of, something we’d push now, because we don’t wear masks in our home.

P9; quality and compliance lead; phase 3

However, we avoided making any further changes to references to masks because we were aware that homes may still use them in the event of another disease outbreak. This was exemplified in phase 4:

I think that’s, yes, giving you the right information. Not necessarily right now. I wouldn’t say that you need to wear a face mask in a care home now, but if there was a virus in the care home which was really contagious as coronavirus was, then that is a good point to have there.

P20; care assistant; phase 4

Overall, there were mixed views on the potential utility of Germ Defence. Most participants felt that the overall concept of Germ Defence was clearly relevant to their context, and some felt that it might be useful as an introduction for new staff members or as a refresher for current staff:

We wouldn’t use it that much...The team have enough to do. They’re really busy. I could imagine that I might use that on orientation, so when I’m getting people in and first starting then I think this is a good introduction to what IPC is all about and then when they’re doing the competencies out on the floor or that’s when they’re actually learning all the practical sides of it.

P4; registered manager and director; phase 3

We know that infection prevention and control can be difficult, sometimes, especially in a busy care home. The Germ Defence website will give you a hand, a refresher, on how to prevent and control the spread of infections. Yeah.

P16; staff nurse; phase 4

However, others felt that they were already doing what Germ Defence suggested and that it did not seem to add anything to their existing IPC training, which was felt to be adequate because inspections had been passed:

I don’t think they’re helpful because I’m already doing it...We’ve had Infection Control round twice and they’ve not pulled anything that I’ve done, so I’m doing my job well...So I wouldn’t...

P17; domestic assistant; phase 4

And if there’s an...like on my phone, probably this morning, I’ve got an update to say that one of the policies has been updated. So, then we will read through it quickly...as things change, we’re kept very much up to date, and I think, I don’t know, I don’t think we need this additionally, really.

P18; chef; phase 4

There were also mixed opinions about the level of detail provided in the information offered by the site. For example, in phase 3, some participants felt that, at certain points, there needed to be comprehensive information to remind them about the transmission of germs and viruses as it is a complex subject:

You need a lot of information to let you know how the infection starts, and how to stop it, and control it, and stuff. If you didn’t have the information, you wouldn’t know.

P1; housekeeper; phase 3

However, at other points, some participants felt that brevity was preferable:

It’s nice and...I like...personally I like a bullet point, so have, like, long...Because of my attention, and other people’s, I think, it needs to be bullet pointed [so it] sticks.

P3; health care assistant (enhanced); phase 3

Participants in phase 3 expressed mixed opinions about the level at which Germ Defence was pitched. Some felt that it was appropriate as it balanced recognition of previous knowledge with a need to be accessible to care staff from a wide range of educational backgrounds:

I think sometimes some other professional bodies tend to dismiss carers as bottom of the rung, which we’re not, it’s a highly professional career now. So, I like that it recognises that we will have some knowledge around this, the infographic is clear, it sets it out very clearly how infection is spread. I think it’s pitched at just the right level really because there are some people who work in care, whose educational background is not as good as others.

P4; registered manager and director; phase 3

However, others felt that it was too basic for experienced staff:

I realise that new staff are coming into an environment and they don’t know things, but I think when you’ve got experienced staff, it sounds a bit, as I say, condescending.

P8; deputy manager; phase 3

As a first attempt to acknowledge this ambivalence, we added a self-rated confidence scale (1=least confident; 10=very confident) to the third page of the website in GDCH version 3 that acknowledged that staff may already be familiar with IPC measures. The page would display 1 of 2 messages depending on the self-rating selection made by the staff member: for lower confidence (1-6), the message would say that Germ Defence would give more advice and guidance about IPC and, for higher confidence (7-10), the message would say that Germ Defence would provide a handy refresher on IPC. In phase 4, feedback on this scale was that some participants felt that staff who rated themselves as very confident about IPC practice would not continue to look through the website:

So that’s just informing us that if we want to look further we can go to that website...Personally, I don’t think many people would look at it...I would say...half the people, they wouldn’t bother.

P18; chef; phase 4

#### Individual Behavior Change in a Complex Environment

These findings relate to the goal-setting mechanism of the intervention, which we modified slightly following stakeholder feedback in phase 2. In phases 3 and 4, participants’ feedback on the goal-setting pages suggested that, although they were easy to read and it was clear how to interact with the pages to answer the questions, it was not clear whether this might lead to any sustained change in behavior. We were initially concerned about the potential for social desirability bias in responses and that participants would simply choose “always” for every behavior. However, this did not appear to be the case as participants appeared to acknowledge shortfalls, at least to some extent:

[Regarding washing hands] touching something that other people have touched, like door handles, and furniture. I'd say, “very often,” but sometimes we might forget, you know what I mean.

P1; housekeeper; phase 3

Some participants appeared to be unconvinced by the “making a plan” goal-setting activity, citing externally driven IPC requirements as having the ultimate influence over individual behavior and highlighting that noncompliance could result in disciplinary action:

Making a plan, yeah, I mean, essentially, we’re wearing an apron and a mask because that is a requirement, so to make the plan of wearing them is what we’re doing anyway because it’s a requirement to do it. So if the aprons came back into being a requirement from IPC, then we wouldn’t be planning on doing it, we’d have to do it, otherwise you wouldn’t be coming to work, you’d have to do it. And the PPE for the personal care is a requirement, and if you don’t do it you’d have to go down the conversations and supervisions and that type of thing.

P6; health and development lead; phase 3

This participant went on to say that the “making a plan” activity may be more beneficial for new, inexperienced colleagues rather than for experienced staff for whom IPC behavior was “embedded.”

Some of the statements regarding the behavior change component specifically related to wearing face masks, which were not mandated at the time of data collection. There were examples to suggest that, if an aspect of IPC was not mandatory, it may be seen as not applicable by some participants:

It doesn’t apply to me but obviously it is optional when you’re giving personal care. But personally, I don’t wear them when it’s not mandatory. So, I’d say “does not apply to me.”

P5; care assistant and domestic team lead; phase 3

There were other examples of how participants’ answers might change depending on the type of activity and area of the home they were thinking about when reading the page:

Face mask, not all the time, these days, but sometimes if we want to get a resident’s room, we are choosing, but in the kitchen, we don’t use it all the time. “Quite often,” maybe.

P14; head chef; phase 4

Therefore, it was unclear whether the questions and possible responses were sensitive enough to the breadth of activities that might arise in the care home setting. Other participants (eg, P12, carer and health care assistant) indicated that they would choose “always” for most questions about their behavior the previous week and would not make any changes to their future plan of behavior. It was unclear whether this response was influenced by social desirability bias. However, some participants appeared more reflective over the exercise, such as one manager who suggested that it made them question their behavior:

It makes you think. So when you have ticked your answers, if they’re not “always” on both sets of the questions, it makes you think about your actions...I clicked “always,” because that’s what I do. But I don’t do it on close contact with a resident, so that was “very often,” because most times I do. Whereas on the second part, and then reading and thinking, that’s maybe one of the places where I should always wash my hands, in close contact with the resident.

P21; registered manager; phase 4

If participants did not indicate substantial improvements to their plan for future activity (ie, if they continued to select answers indicating a low frequency of behavior), they would then see a page asking them to reconsider. Some suggested that they felt indifferent about this page—there was nothing “wrong with it,” but it did not appear to be very motivating:

Interviewer: So, now you have this page saying “are you happy with your plan?” What do you think of this?

Participant: I don’t really think anything of it. I don’t know...sanitise your hands sometimes before coming into close contact with a care home resident, why not try wash and sanitising them quite often from now on. Yes, I won’t say anything’s wrong with it.

P20; care assistant; phase 4

There were also some comments about the utility of conceiving the goal-setting exercise across a weekly time frame, how the intervention might be implemented alongside the remit of infection control leads, and whether there needed to be some output that captured the plan that a staff member had made as “evidence” of their proposed change in behavior:

[It would be useful if it was] downloadable or we can save it in some way to build it into the audit for us, as an evidence-base...we wouldn’t do it weekly, they get so many competency checks and supervisories and everything else that this would be yet another thing we would add in. But it’s certainly something we could add in as part of that auditing process that we do which is done at a minimum of twice yearly...Then if we find somebody isn’t following procedure or isn’t changing the PPE effectively, then we can go back and go, “look, you said you were going to do it, so do it,” and just have that evidence-base there a little bit. I think but because we keep on top of it and because we’ve got infection leads, it’s not something we would do weekly by any stretch. I think you would be hard pressed to find a home that would do it weekly.

P4; registered manager and director; phase 3

Therefore, overall, our findings suggest that, while the goal-setting behavior change mechanism may have potential, more work would be needed to refine it to facilitate successful individual behavior change when there is organizational-level regulation about what IPC actions should be taken by staff and how often.

## Discussion

### Principal Findings

This paper reports on qualitative work to evaluate whether and how an existing web-based, evidence-based behavior change IPC intervention called Germ Defence might be adapted to meet the needs of care home staff. Overall, our findings showed that adapting Germ Defence to this context is extremely challenging. Participants had mixed views about the appeal of Germ Defence and its potential suitability to the care home context and suggested that much more work would need to be done to make Germ Defence more engaging, accessible, and relevant to the care home workforce. The original Germ Defence intervention was designed for a very different population with little IPC knowledge. The elements that explained the need for IPC tended to duplicate existing training, and the goal setting for IPC actions did not appear to make sense to care home staff, whose primary concern may have been to follow mandatory IPC requirements. Following completion of our care home study, the results of the large cluster-randomized controlled trial of the COVID-19 adaption of Germ Defence in English general practice underway at the same time [42] showed no evidence that the intervention affected rates of respiratory tract infections or other health outcomes [51]. As the actual use of the intervention was below the 25% assumed in the sample size calculations, it is difficult to draw robust conclusions [51]. However, the results of this trial are important to note in the context of our study. Our findings suggest that substantial further work would be required to adapt Germ Defence to the care home context.

As qualitative descriptive approaches do not aim to provide in-depth theoretical analysis [52], it is useful to offer some brief discussion of our findings by drawing upon the widely used theoretical model of the Unified Theory of Acceptance and Use of Technology (UTAUT) [53]. The UTAUT’s unit of analysis is the individual user, which is applicable for our study because our aim was to work with individual members of care home staff to try to adapt Germ Defence to their occupational context [54]. Although most commonly used quantitatively, the UTAUT has been used as a qualitative analytical and interpretive lens (eg, a recent qualitative meta-synthesis of digital interventions for antimicrobial prescribing and monitoring [55]). The UTAUT comprises 4 main constructs; our data speak mostly to the first 2 that focus on perceptions of benefit and ease of use.

*Performance expectancy* refers to the degree to which participants believe that using the intervention would be beneficial. Our findings within this construct appear to be mixed. Some respondents felt that Germ Defence could be potentially useful to support induction of new staff or provide refresher training, but others suggested that it may be too simplistic. The degree to which participants thought that it would offer a genuine benefit was unclear. Although we did not explicitly investigate the current IPC training provided in each care home in depth, some participants’ responses suggested that more work would need to be done for Germ Defence to be seen as a useful addition to their existing training and practice.

*Effort expectancy* refers to the degree of ease of use of the intervention. Again, our findings within this construct were mixed, with some participants talking positively about the simple layout but others finding it confusing. Many participants suggested that the images would need substantial refinement as they did not always clearly convey the message of the text. Feedback also suggested that a text-heavy website would not be suitable because staff may be too busy to read through it all, may prefer audio or video content, or may prefer more practical training. Although the goal-setting exercises appeared to be easy to understand in principle, an investigation into their effectiveness for behavior change was beyond the scope of this study. We did not have the resources to develop multimedia content (eg, video or audio options) to assess whether it would be engaging enough to result in repeated, realistic practice of the desired behaviors.

Although the reasons for this low take-up are unclear, we note that Germ Defence was initially developed approximately 15 years before this work, and in the context of care homes, it is unclear from our study whether further context-specific adaptations would be sufficient to deliver engaging content that meets contemporary expectations. For instance, the potential of virtual reality (VR) and augmented reality to deliver engaging and realistic training in technical medical skills has long been recognized, and there is increasing recognition of their potential to support training in nonclinical skills [56-58]. Recent work exploring the development and use of VR and augmented reality hand hygiene training with care home staff has found that staff were extremely positive about immersive VR delivery, perceiving it to be more engaging and enjoyable than previous training and valuing its inherent repeated practice, task realism, and feedback inherent to the intervention [59]. However, there are some challenges with this mode of delivery (eg, some side effects of nausea from using VR that render it unsuitable for all and up-front cost implications) [59].

The other 2 constructs of the UTAUT refer to external factors influencing implementation of the intervention. The focus of our study meant that we had less data relevant to these constructs. *Social influence* captures the degree to which participants perceive that important others believe that they should use the intervention. Although our interviews did not explicitly focus on this construct, some participants suggested that external directives regarding IPC from authoritative bodies would be a stronger driver of behavior than individual goal setting. However, several studies suggest that adherence to external directives is suboptimal [21,22,26]. Therefore, Germ Defence could be designed to improve adherence to IPC rules and guidance for care homes and explain when certain IPC measures are useful even if they are not mandatory (eg, explaining why mask wearing reduces transmission of respiratory infections when a staff member or resident has a cough). Finally, *facilitating conditions* highlights the degree to which participants feel that their organization’s infrastructure would support the intervention’s use. Although these kinds of implementation questions were not addressed directly in our study, some participants suggested that Germ Defence would need to be seen as compatible with their routine use of smartphones and tablets. Some managers suggested that Germ Defence could be used alongside IPC audits, suggesting that there is potential support from care homes’ administrative infrastructure. These issues warrant further consideration.

### Strengths and Limitations

The main strength of this study is its diverse sample of organizations and participants. We recruited care homes across the spectrum of quality rating according to the sector regulator and homes providing both residential only and nursing care. We did not observe any strong differences in views across types of homes, suggesting that our findings are likely to apply across the care home sector. Within the homes, we recruited participants working in a wide range of roles, including senior management, direct care provision, and ancillary positions, and with a wide range of experience, from a few months to >2 decades. Some of our findings highlight possible differences in views from staff in different roles (eg, the questionable relevance of some of the current content to ancillary staff and managers’ views on the potential of the intervention to be used to demonstrate compliance), which would warrant further consideration in intervention development. Therefore, we believe that our findings are likely to have a high degree of transferability to the care home sector more widely. In addition, we provided a transparent description of our study methods, including the iterative phases of the study, to promote credibility and dependability.

The main limitation of our study is its relatively rapid nature. It was conceived in response to the crisis faced in the care home sector from the COVID-19 pandemic, with the aim to consider how an existing evidence-based intervention might be adapted to meet the needs of care homes. The think-aloud interviews to capture participants’ real-time reactions to using Germ Defence were not designed to elicit detailed perspectives on potential facilitators of and challenges to implementation of the intervention that may contribute to intervention design. Our data lend themselves to a descriptive summary analysis rather than to a more nuanced exploration via a more interpretive approach, such as thematic analysis [60]. Despite these limitations, our study highlights the challenges of making rapid adaptations to Germ Defence in this context. Any such work would require a more sophisticated methodological approach, drawing more comprehensively on implementation theory. This could involve a greater focus on more fundamental aspects of intervention design advocated at an earlier stage of the person-based approach methodology [46], including a detailed investigation of current IPC training and practice and more detailed, theoretically informed exploration of participants’ understandings of the intervention (eg, via a greater focus on the constructs of the UTAUT or of normalization process theory [61]). Such an approach was beyond the scope of this study. However, it is also unclear whether such work would be merited. Finally, it is worth noting that the ongoing independent public inquiry into the UK government’s decision-making during the pandemic includes scrutiny on the extent to which care homes were protected and highlights that, in addition to single interventions, there is an urgent need for well-prepared, coherent, strategic IPC responses to such health emergencies across health and care systems [62,63].

### Conclusions

Our study highlights the challenges of rapidly adapting an existing intervention to a new context. Future research in this area would require a pragmatic methodological approach with a focus on implementation. In the context of the GDCH intervention, it is unclear whether such work is warranted.

## Abbreviations

GDCH: Germ Defence for Care Homes
IPC: infection prevention and control
PPE: personal protective equipment
PRIMIT: Primary Care Trial of a Website-Based Infection Control Intervention to Modify Influenza-like Illness and Respiratory Infection Transmission
UTAUT: Unified Theory of Acceptance and Use of Technology
VR: virtual reality

## Data Availability

The datasets generated or analyzed during this study are available from the corresponding author on reasonable request.
